# ANXA2, PRKCE, and OXT are critical differentially genes in Nonalcoholic fatty liver disease 

**Published:** 2019

**Authors:** Mostafa Rezaei Tavirani, Majid Rezaei Tavirani, Mona Zamanian Azodi

**Affiliations:** 1 *Proteomics Research Center, Faculty of Paramedical Sciences, Shahid Beheshti University of Medical Sciences, Tehran, Iran*; 2 *Faculty of Medicine, Iran University of Medical Sciences, Tehran, Iran *

**Keywords:** Nonalcoholic fatty liver disease, Biomarker, Gene

## Abstract

**Aim:**

Identification of prominent genes which are involved in onset and progress of steatosis stage of Nonalcoholic fatty liver disease (NAFLD) is the aim of this study.

**Background:**

NAFLD is characterized by accumulation of lipids in hepatocytes. The patients with steatosis (the first stage of NAFLD) will come across nonalcoholic steatohepatitis (NASH) and finally hepatic cirrhosis. There is correlation between cirrhosis and hepatic cancer. However, ultrasonography is used to diagnose NAFLD, biopsy is the precise diagnostic method.

**Methods:**

Gene expression profiles of 14 steatosis patients and 14 controls are retrieved from gene expression omnibus (GEO) and after statistical validation top 250 differentially expressed genes (DEGs) were determined. The characterized DEGs were included in network analysis and the central DEGs were identified. Gene ontology (GO) performed by ClueGO analysis of DEGs to determine critical biological terms. Role of prominent DEGs in steatosis is discussed in details.

**Results:**

Numbers of 31 significant DEGs including 20 up-regulated and 11 down-regulated ones were determined. Nine biological groups including 27 terms were recognized. Negative regulation of low-density lipoprotein particle receptor catabolic process, TRAM-dependent toll-like receptor signaling pathway, and regulation of hindgut contraction which were related to ANXA2, PRKCE, and OXT respectively were determined as critical biological term groups and DEGS.

**Conclusion:**

Deregulation of ANXA2, PRKCE, and OXT is a critical event in steatosis. It seems these three genes are suitable biomarker to diagnosis of steatosis.

## Introduction

 Accumulation of lipids in hepatocytes occurs in NAFLD which is seen in association with various diseases, toxins, and drugs. There is evidence that level hepatic enzymes change significantly. Steatosis is the first stage of NAFLD which can convert to nonalcoholic steatohepatitis (NASH) and finally cirrhosis (1, 2). There is correlation between cirrhosis and liver cancer (3). Usually, ultrasonography check of liver is diagnostic method for NAFLD. However, precious diagnosis requires liver biopsy (4). Several attempts are done to find noninvasive biomarkers for NAFLD. Proteomics, genomics, metabolomics, and bioinformatics are used to analyze molecular aspect of NAFLD (5-8). In one study cytokeratin-18 fragment level is introduced as noninvasive biomarker for NAFLD while in the other investigation a large number of biomarkers are tabulated and introduced to diagnose NAFLD (9, 10). 

PPI network analysis is used to interact large numbers of proteins (or genes) in a network to provide possible screening tool. In this approach few genes among interacted genes play critical role to construct the network, and therefore have many connection with the other elements of the network. These types of genes are known as hubs. The other types of important elements of network are known as bottlenecks. The common hubs and bottlenecks are famous as hub-bottlenecks (11, 12). Gene ontology is useful method to identify biological terms related to investigate genes. In this regard, molecular function, biological processes, cellular component, and biochemical pathways as molecular features related to the query genes recognize (13). In this study, gene expression profiles of NAFLD (steatosis stage) patients are compared with control (data are retrieved from GEO) and the significant DEGs are included into interactome to find critical genes which are involved in NAFLD. Gene ontology is used to identify biological terms related to NAFLD. The finding can be considered to determine possible diagnostic and therapeutic biomarkers. 

## Methods

Gene expression profiles of 14 non-alcoholic fatty liver disease (NAFLD) stage steatosis patients and 14 controls, series GSE48452, GPL11532 were obtained from GEO. Demography of samples are tabulated in the table 1 (14). Liver biopsy samples were analyzed by array-based DNA methylation and mRNA expression. Profiles were compared via boxplot analysis and top 250 significant DEGs were considered for more analysis. The characterized DEGs with fold change more than 1.5 were determined and included in PPI network. The query DEGs and 100 added relevant genes were included in network to constructed PPI network by using STRING database and Cytoscape software (15, 16). The network was analyzed via Network analyzer plugin of Cytoscpe software. Biological terms relative to the query DEGs were determined and clustered by ClueGO (17). Critical DEGs based on network analysis and GO enrichment were determined. For more understanding a PPI network including the critical DEGs and their direct neighbors was built. 

## Results

**Figure 1:**
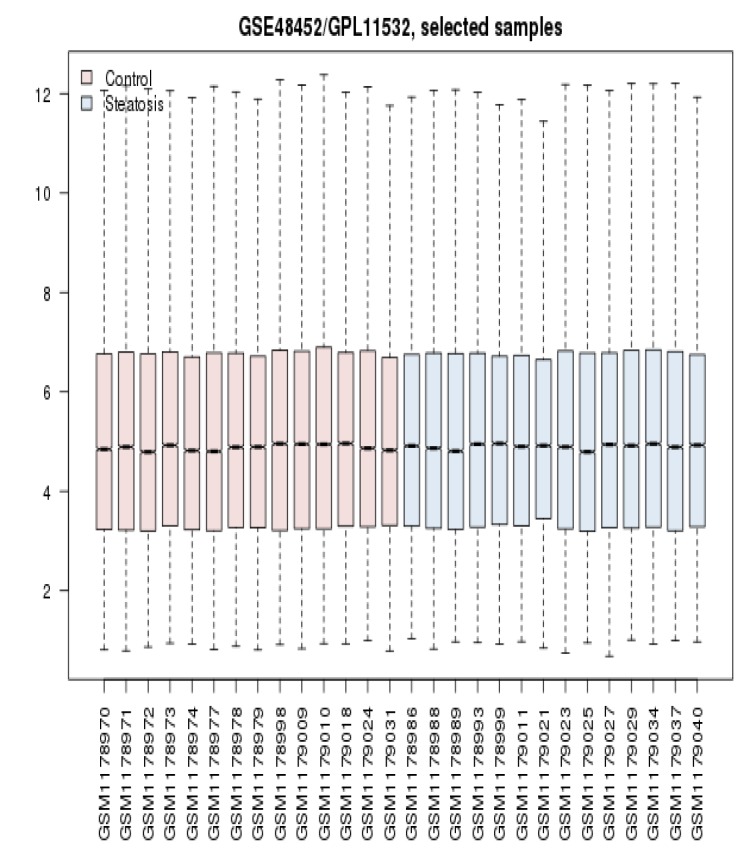
Boxplot presentation of gene expression profiles of 14 steatosis patients and 14 controls

Gene expression profiles of 14 steatosis samples and 14 controls are analyzed via boxplot analysis. As it is shown in the figure 1 data obey from median centered distribution, therefore they are statistically comparable. 

**Figure 2:**
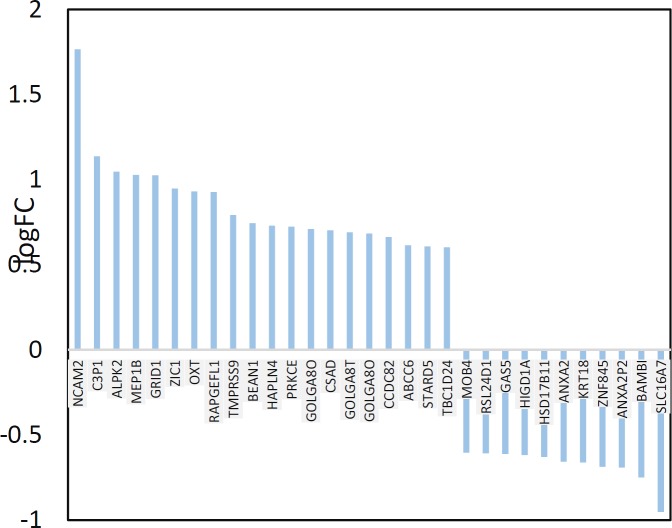
LogFC 31 DEGs including 20 up-regulated and 11 down-regulated ones is illustrated

**Figure 3:**
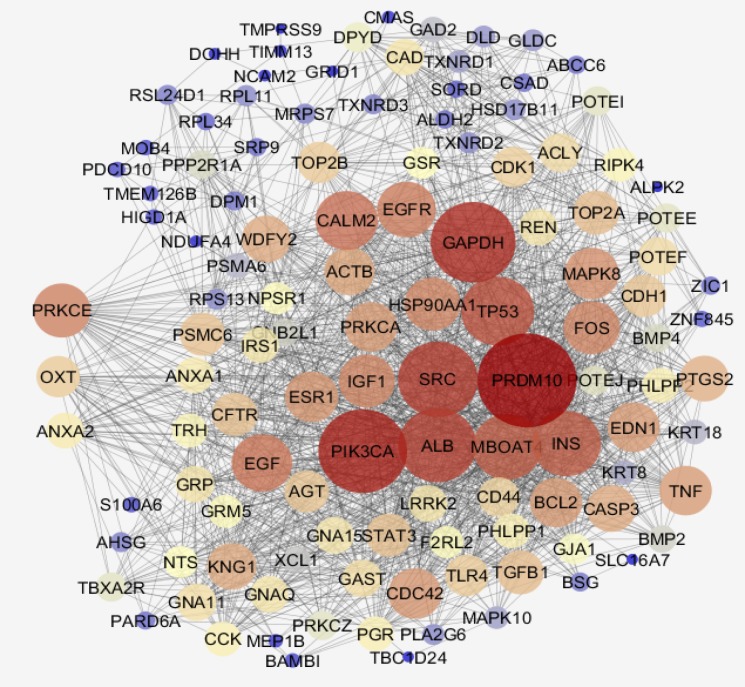
The main connected component including 120 nodes and 1576 edges is shown. The nodes are layout based on degree value. PRKCE, OXT, and ANXA2, the query DEGs are shown in the left side of figure

Among 250 top significant DEGs, 31 individuals were characterized and included in network construction. In figure 2, the 31 DEGs and their LogFC are shown. In this figure, it is appeared that there are 20 up – regulated and 11 down – regulated DEGs. The network was built by 31 query DEGs and 100 added relevant ones. As it is illustrated in figure 3, 4 DEGs were not recognized and the network was constructed by 127 nodes and 1576 edges. 

**Figure 4:**
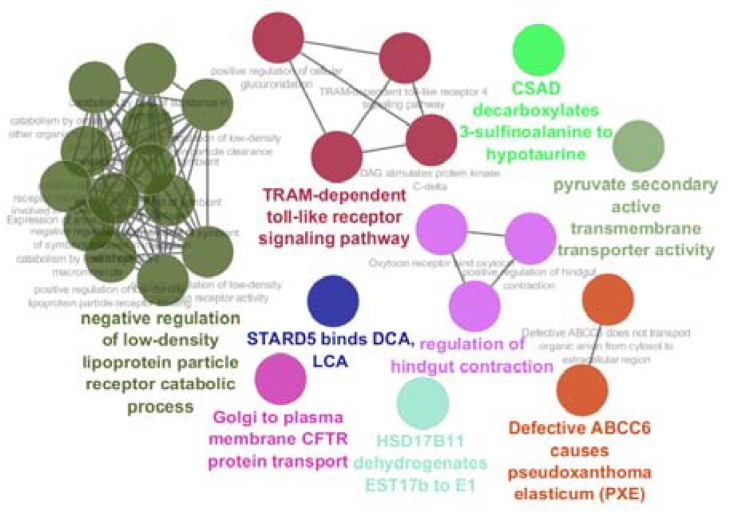
Nine groups including 27 terms relevant to the 31 DEGs are shown

**Figure 5:**
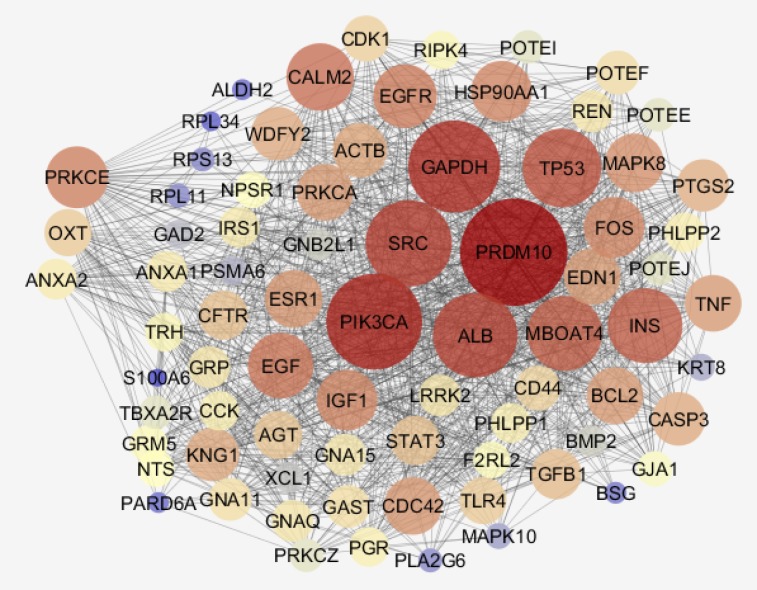
Network including PRKCE, OXT, and ANXA2 and their direct neighbors is illustrated. The nodes are layout based on degree value. Bigger size is corresponded to high value of degree. Color from blue to red refers to increment of degree value

Numbers of 7 nodes were isolated and the main connected component contains 120 nodes. Biological terms relative to the 31 DEGs are presented in the figure 4. The 27 terms are grouped in 9 classes. Details of figure 4 and additional information are tabulated in table 2. As it is shown in this table only 9 genes among 31 DEGs are involved in the biological terms. As it is shown in the figure 3 and table 2 three important DEGs are included PRKCE, OXT, and ANXA2. These three DEGs and their direct neighbors are shown in figure 5. 

**Figure 6:**
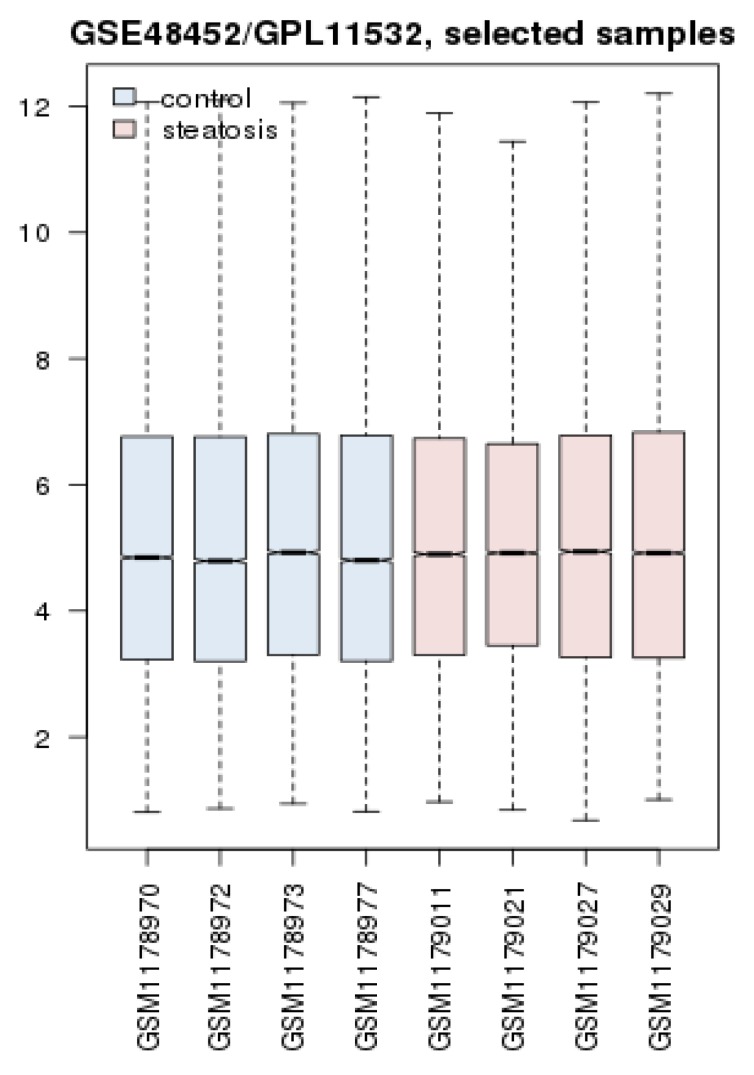
Boxplot analysis of gene expression profiles of 4 control in comparison with 4 steatosis patients samples

Considering important role of oxytocin in our study and well-known function of this hormone in females, expression change of OXT in male patients and control was investigated. Therefore, possible bias of sex effect is considered. It was appeared that fold change of oxytocin was equal to 3.76 (LogFC = 1.91) and this hormone was the top deregulated DEGs. Demography of samples and boxplot analysis are shown in table 3 and figure 6.

**Table 1:** Demography of controls and patients    (14)

R	Accession	Group	Sex	Age	Bmi
1	GSM1178970	Control	male	53	25.8
2	GSM1178971	Control	female	51	23.6
3	GSM1178972	Control	male	77*	26.9*
4	GSM1178973	Control	male	23	26.6
5	GSM1178974	Control	male	80	25.8
6	GSM1178977	Control	male	68	26.4
7	GSM1178978	Control	female	45	20.1
8	GSM1178979	Control	female	44	29.4
9	GSM1178986	Steatosis	female	46	43.5
10	GSM1178988	Steatosis	female	24	51.9
11	GSM1178989	Steatosis	female	32	48.6
12	GSM1178993	Steatosis	female	38*	42.4*
13	GSM1178998	Control	female	28	17.4
14	GSM1178999	Steatosis	female	47	56
15	GSM1179009	Control	female	38*	30.0*
16	GSM1179010	Control	female	42	23.3
17	GSM1179011	Steatosis	male	61	40.3*
18	GSM1179018	Control	female	73	21
19	GSM1179021	Steatosis	male	38*	55.5*
20	GSM1179023	Steatosis	female	33	40.9
21	GSM1179024	Control	female	44	24.9
22	GSM1179025	Steatosis	female	39*	53.6*
23	GSM1179027	Steatosis	male	47*	47.9*
24	GSM1179029	Steatosis	male	65	43.7
25	GSM1179031	Control	female	60*	30.8*
26	GSM1179034	Steatosis	female	42	49.6
27	GSM1179037	Steatosis	female	32	60.2
28	GSM1179040	Steatosis	female	39*	41.8*

**Table 2:** Numbers of 27 terms relevant to the 31 DEGs are clustered in the 9 groups. %G/T refers to percentage of genes that are involved in term. Gene column shows the gene which participates in the related term

R	GO Term	Ontology Source	Group	% G/T	Gene
1	**pyruvate secondary active transmembrane transporter activity**	GO_MolecularFunction-EBI-QuickGO-GOA_20.11.2017_00h00	1	100	SLC16A7
2	**CSAD decarboxylates 3-sulfinoalanine to hypotaurine**	REACTOME_Reactions_20.11.2017	2	100	CSAD
3	**HSD17B11 dehydrogenates EST17b to E1**		3	100	HSD17B11
4	**STARD5 binds DCA, LCA**		4	100	STARD5
5	**Golgi to plasma membrane CFTR protein transport**	GO_BiologicalProcess-EBI-QuickGO-GOA_20.11.2017_00h00	5	50	KRT18
6	**Defective ABCC6 causes pseudoxanthoma elasticum (PXE)**	REACTOME_Pathways_20.11.2017	6	100	ABCC6
7	Defective ABCC6 does not transport organic anion from cytosol to extracellular region	REACTOME_Reactions_20.11.2017			
8	Oxytocin receptor bind oxytocin		7	50	OXT
9	**regulation of hindgut contraction**	GO_BiologicalProcess-EBI-QuickGO-GOA_20.11.2017_00h00			
10	positive regulation of hindgut contraction			100	
11	DAG stimulates protein kinase C-delta		8	50	PRKCE
12	**TRAM-dependent toll-like receptor signaling pathway**				
13	TRAM-dependent toll-like receptor 4 signaling pathway				
14	positive regulation of cellular glucuronidation			100	
15	Expression of annexin A2	REACTOME_Reactions_20.11.2017	9	50	ANXA2
16	negative regulation of development of symbiont involved in interaction with host	GO_BiologicalProcess-EBI-QuickGO-GOA_20.11.2017_00h00		100	
17	positive regulation of low-density lipoprotein particle clearance				
18	catabolism by organism of protein in other organism involved in symbiotic interaction				
19	catabolism by host of substance in symbiont				
20	metabolism by host of symbiont macromolecule				
21	metabolism by host of symbiont protein				
22	**negative regulation of low-density lipoprotein particle receptor catabolic process**			50	
23	catabolism by host of symbiont macromolecule			100	
24	positive regulation of low-density lipoprotein particle receptor binding				
25	positive regulation of low-density lipoprotein receptor activity				
27	catabolism by host of symbiont protein				
27	positive regulation of receptor-mediated endocytosis involved in cholesterol transport			50	

**Table 3:** Demography of 4 male patients and 4 male controls which their gene expression profiles are compared is shown

R	Accession	Group	Sex	Age	Bmi
1	GSM1178970	Control	male	53	25.8
3	GSM1178972	Control	male	77*	26.9*
4	GSM1178973	Control	male	23	26.6
6	GSM1178977	Control	male	68	26.4
17	GSM1179011	Steatosis	male	61	40.3*
19	GSM1179021	Steatosis	male	38*	55.5*
23	GSM1179027	Steatosis	male	47*	47.9*
24	GSM1179029	Steatosis	male	65	43.7

## Discussion

Gene profile analysis can provide useful information about molecular mechanism of diseases (18, 19). In this study network analysis is used to screen significant DEGs which differentiated steatosis stage of NAFLD patients from controls. As it is shown in the figure 1, statistically samples are comparable because distribution of data is median centric. Therefore, more investigations about samples are possible. As it is shown in figure 2, up-regulation is prominent relative to down-regulation in NAFLD. Numbers of 20 DEGs are up-regulated while 11 down-regulated genes are represented. However, PRDM10, PIK3CA, GAPDH, ALB, SRC, and TP53 are the hubs of the network but PRKCE, OXT, and ANXA2 the query DEGs play significant roles in the PPI network. PRKCE and OXT are upregulated genes while ANXA2 is down regulated one (see figure 2). In figure 3, positions of these three DEGs relative to the other query DEGs are layout and illustrated. The other query DEGs are characterized with weak centrality role in the network. Among 31 DEGs, seven ones containing BEAN1, CCDC82, GOLGA8O, GOLGA8T, HAPLN4, RAPGEFL1, and STARD5 were not included in the network and remained as isolated nodes. GO analysis indicates that 9 DEGs are involved in the 27 biological terms which are clustered in the 9 groups (see figure 4 and table 2). Group 9: negative regulation of low-density lipoprotein particle receptor catabolic process is the largest group including 13 biological terms. The second and third larger groups (groups 8 and 7) are TRAM-dependent toll-like receptors signaling pathway (including 4 terms) and regulation of hindgut contraction (containing 3 terms), respectively. ANXA2, PRKCE, and OXT are complicated in the groups 9, 8, and 7, respectively. These three DEGs are involved in 20 terms (74% of all biological Terms). As it is shown in the figure 5, 77 nodes (64% of PPI network nodes) are directly linked to PRKCE, OXT, and ANXA2. It seems ANXA2, PRKCE, and OXT are central DEGs among 31 query DEGs which their deregulation is functionally significant event in NAFLD. 

Since oxytocin is a well-known female hormone and there are no sufficient documents about its role in men, we design another analysis that was resulted from comparison between male patients and control (see table 3 and figure 6). In this analysis OXT appeared as the top DEGs based on fold change. Therefore, presence of oxytocin among three important DEGs in NAFLD is depended to both male and female patients. 

Four biological terms in group 9 are about regulation of low-density lipoprotein (LDL). LDL is a cholesterol-carrying agent in human plasma which LDL receptor regulates its plasma level. Investigation showed that raising cholesterol content of liver hepatocytes leads to fall of LDL receptors in liver which causes increment of LDL level of plasma. This process is seen after digestion of diets rich in saturated fat and cholesterol (20). It seems that deregulation of ANXA2 effects on storage of fat in lever via deregulation of clearance of plasma cholesterol. Sun *et al*. reported that there is correlation between low-density lipoprotein cholesterol and NAFLD prevalence (21). However, role of ANXA2 in NAFLD is reported previously, here it is introduced as the top related gene in NAFLD (especially steatosis stage).

Positive regulation of cellular glucuronidation is one of group 8 biological process. Glucuronidation is a major biochemical pathway that plays role in cellular detoxification. In this pathway, the highly hydrophilic glucuronide group transfers to hydrophobic substrates which are less toxic and can be exerted easily relative to the initial substances (22). Role of this protective process was studied in drug metabolism of NAFLD patients (23). It can be concluded that up-regulation of PRKCE which promotes positive regulation of cellular glucuronidation is a protecting activity in NAFLD. 

Regulation of hindgut contraction is a term which is related to oxytocin. It is reported that positive regulation of hindgut (the posterior part of the alimentary canal, including the rectum, and the large intestine) is appositive regulation of smooth muscle which positively regulates hindgut contraction. This terms is responsible for positive regulation of digestion (https://www.ebi.ac.uk/QuickGO/term/GO:0060450). In conclusion up-regulation of OXT stimulates digestion in steatosis stage of NAFLD. 

Precious analysis revealed that ANXA2, PRKCE, and OXT are three important genes that are involved in steatosis stage of NAFLD. Significant expression change, participation in prominent biochemical pathways, and large number of connections with the other genes imply that these DEGs be considered as critical genes relative to NAFLD. It can be suggested that suitable quantity profiles of ANXA2, PRKCE, and OXT be validated to manage steatosis stage of NAFLD.
